# Dihydrolipoic Acid Induces Cytotoxicity in Mouse Blastocysts through Apoptosis Processes

**DOI:** 10.3390/ijms13033988

**Published:** 2012-03-22

**Authors:** Wei-Li Houng, Cheng-An J. Lin, Ji-Lin Shen, Hung-I Yeh, Hsueh-Hsiao Wang, Walter H. Chang, Wen-Hsiung Chan

**Affiliations:** 1 Department of Bioscience Technology and Center for Nanotechnology, Chung Yuan Christian University, Chung Li 32023, Taiwan; E-Mail: sylvie19830406@hotmail.com; 2 Department of Biomedical Engineering and Center for Nano Bioengineering, Chung Yuan Christian University, Chung-Li 32023, Taiwan; E-Mail: chengan_lin@cycu.edu.tw (C.-A.J.L.); 3 Department of Physics, Chung Yuan Christian University, Chung Li 32023, Taiwan; E-Mail: jlshen@phys.cycu.edu.tw; 4 Departments of Internal Medicine and Medical Research, Mackay Memorial Hospital, Mackay Medical College, New Taipei City 252, Taiwan; E-Mails: hiyeh@ms1.mmh.org.tw (H.-I.Y.); okul.wang@gmail.com (H.-H.W.)

**Keywords:** dihydrolipoic acid, blastocyst, apoptosis, embryonic development

## Abstract

α-Lipoic acid (LA) is a thiol with antioxidant properties that protects against oxidative stress-induced apoptosis. LA is absorbed from the diet, taken up by cells and tissues, and subsequently reduced to dihydrolipoic acid (DHLA). In view of the recent application of DHLA as a hydrophilic nanomaterial preparation, determination of its biosafety profile is essential. In the current study, we examined the cytotoxic effects of DHLA on mouse embryos at the blastocyst stage, subsequent embryonic attachment and outgrowth *in vitro*, *in vivo* implantation by embryo transfer, and early embryonic development in an animal model. Blastocysts treated with 50 μM DHLA exhibited significantly increased apoptosis and a corresponding decrease in total cell number. Notably, the implantation success rates of blastocysts pretreated with DHLA were lower than that of their control counterparts. Moreover, *in vitro* treatment with 50 μM DHLA was associated with increased resorption of post-implantation embryos and decreased fetal weight. Data obtained using an *in vivo* mouse model further disclosed that consumption of drinking water containing 100 μM DHLA led to decreased early embryo development, specifically, inhibition of development to the blastocyst stage. However, it appears that concentrations of DHLA lower than 50 μM do not exert a hazardous effect on embryonic development. Our results collectively indicate that *in vitro* and *in vivo* exposure to concentrations of DHLA higher than 50 μM DHLA induces apoptosis and retards early pre- and post-implantation development, and support the potential of DHLA to induce embryonic cytotoxicity.

## 1. Introduction

α-Lipoic acid (LA), a type of thioctic acid, is naturally synthesized by some plants and animals, including humans [1]. Endogenous LA acts as a cofactor for important mitochondrial enzymes and other multienzyme complexes, including those of branched-chain α-keto acid, pyruvate dehydrogenase, α-ketoglutarate dehydrogenase, and glycine decarboxylase [2,3]. LA absorbed from food intake crosses the blood-brain barrier, is transported and taken up by cells, and subsequently converted to the reduced form, dihydrolipoic acid (DHLA) [4]. Although LA and DHLA display pro-oxidant properties under specific conditions, both compounds additionally act as strong antioxidants [5,6]. Recent studies showed that LA and DHLA possess anti- or proapoptotic properties. These compounds function in various cell types to block or prevent oxidative stress-induced apoptosis but promote apoptosis in several cancer cell lines [7–12]. In addition, our recent study showed that DHLA (50–100 μM) induces apoptotic processes in mouse embryonic stem cells (ESC-B5) [13]. DHLA (50–100 μM) directly increased the reactive oxygen species (ROS) content in ESC-B5 cells, along with a significant increase in cytoplasmic free calcium and nitric oxide (NO) levels, loss of mitochondrial membrane potential (MMP), activation of caspases-9 and -3, and cell death. Our results collectively indicate that DHLA at concentrations of 50–100 μM triggers apoptosis of ESC-B5 cells, which involves both ROS and NO [13]. The ambiguous issue of whether LA and DHLA are pro-/antioxidant or pro-/antiapoptosis agents requires further investigation. DHLA is an important capping ligand utilized in the preparation of nanoparticles for cellular labeling and tracking, including detection of tracers in embryonic development investigation [14–16]. Thus, it is crucial to determine the safety and toxicity of DHLA, both in cells and embryonic development.

Apoptosis plays an important role in development and disease [17]. While several studies have shown that apoptosis functions in normal embryonic development [18–20], mechanistically diverse teratogens can induce excessive apoptosis in early embryos, leading to developmental injury [21–25]. Previous studies have demonstrated that DHLA induces apoptosis in mammalian cells, including lung cancer and HL-60 leukemia cells [12,26]. In the present study, we further investigated whether the DHLA has cytotoxic effects on embryonic development, using mouse blastocysts as the assay model. Our results showed that DHLA suppresses embryonic cell proliferation during the blastocyst stage predominantly via inducing apoptosis in the inner cell mass (ICM), but has no effect on the trophectoderm (TE). The effects of DHLA on subsequent developmental injury of blastocysts *in vitro* and embryo transfer *in vivo* were additionally examined.

## 2. Results

### 2.1. Effects of DHLA on Mouse Blastocysts

**Figure 1:**
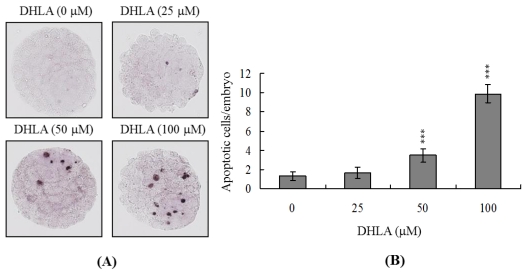
Dihydrolipoic acid (DHLA) induces apoptosis in mouse blastocysts. (**A**) Mouse blastocysts were treated with DHLA (25, 50 or 100 μM) for 24 h or left untreated, and apoptosis examined via TUNEL staining. Cells were visualized using light microscopy. TUNEL-positive cells are depicted in black; (**B**) The mean number of apoptotic (TUNEL-positive) cells per blastocyst was calculated. Values are presented as means ± SEM of ten determinations. *** *P* < 0.001 *versus* the control group.

To investigate the possibility of DHLA-induced cytotoxicity, we treated mouse blastocysts with 25, 50 or 100 μM DHLA at 37 °C for 24 h, and monitored apoptosis using the TUNEL method. Cellular apoptosis was evident in blastocysts treated with 50 μM DHLA (Figure 1A). Quantitative analysis revealed ~2.5 to 7.5-fold more apoptotic cells in 50–100 μM DHLA-treated blastocysts, compared with untreated control cells (Figure 1B). Clearly, DHLA induces apoptosis in mouse blastocysts within the 50–100 μM concentration range.

### 2.2. Effects of DHLA on Cell Proliferation

**Figure 2:**
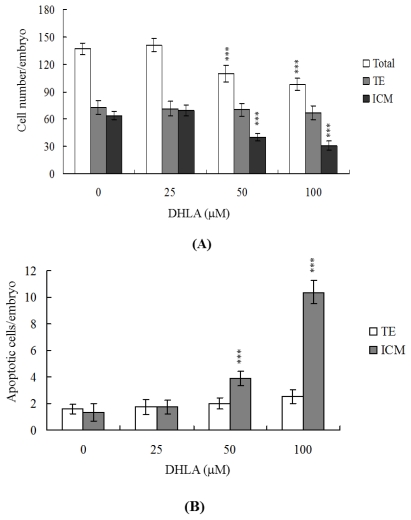
Effects of DHLA on blastocyst viability. Mouse blastocysts were treated with or without DHLA (25, 50 or 100 μM) for 24 h. (**A**) The total number of cells per blastocyst and cell numbers in the inner cell mass (ICM) and trophectoderm (TE) were counted; (**B**) The percentages of Annexin V-positive/PI-negative cells in blastocysts of each group were examined. Data are based on at least 200 blastocyst samples from each group. *** *P* < 0.001 *versus* the control group.

Differential staining, followed by cell counting, was used to assess cell proliferation in blastocysts either treated with 25, 50 or 100 μM DHLA for 24 h or left untreated. We observed significantly lower cell numbers in 50 μM DHLA-treated blastocysts, compared with control cells (Figure 2A). Annexin V staining revealed markedly higher numbers of Annexin V-positive/PI-negative (apoptotic) cells in the ICM of treated blastocysts *versus* controls, but no such differences in the trophectoderm (TE) (Figure 2B). Our experiments show that 50–100 μM DHLA induces significant apoptosis in the ICM, but not TE, of mouse blastocysts, further supporting the theory that DHLA impairs the developmental potential of blastocysts.

### 2.3. Effects of DHLA on Mouse Embryonic Developmental Potential *in Vitro*

**Figure 3:**
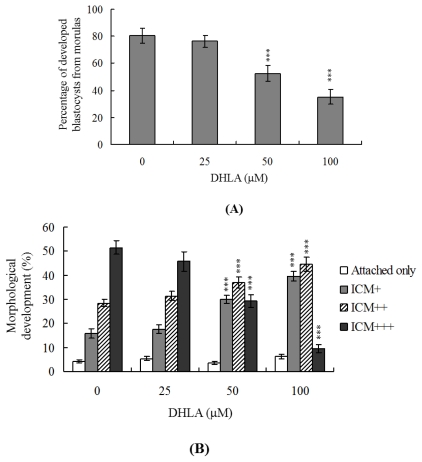
*In vitro* development of mouse embryos exposed to DHLA at the blastocyst stage. (**A**) Mouse morulae were treated with DHLA (25, 50 or 100 μM) for 24 h or left untreated, and cultured for an additional 24 h at 37 °C. Blastocysts were counted and percentages calculated; (**B**) Mouse blastocysts were treated with DHLA (25, 50 or 100 μM) for 24 h or left untreated and cultured for 7 days post-treatment. Blastocysts were identified as attachment only, ICM(+), ICM(++), and ICM(+++) via morphological assessment, as described in Materials and Methods. Values are presented as means ± SEM of eight determinations. *** *P* < 0.001 *versus* the control group.

Untreated control morulae displayed 80% development to blastocysts, whereas only 52% of those treated with 50 μM DHLA developed into blastocysts under our experimental conditions (Figure 3A). To further determine the effects of DHLA on post-implantation events *in vitro*, we treated blastocysts with or without 25, 50 or 100 μM DHLA, and analyzed subsequent development for 8 days in culture. Importantly, the rate of embryo attachment to fibronectin-coated culture dishes and lack of further development (attachment only group) has no any effects by treatment with DHLA (Figure 3B). However, DHLA-pretreated blastocysts displayed a lower incidence of post-implantation developmental milestones (Figure 3B). Our results clearly indicate that DHLA affects the *in vitro* potential of blastocysts to develop into post-implantation embryos.

### 2.4. Effects of DHLA on the Developmental Potential of Blastocysts *in Vivo*

**Figure 4:**
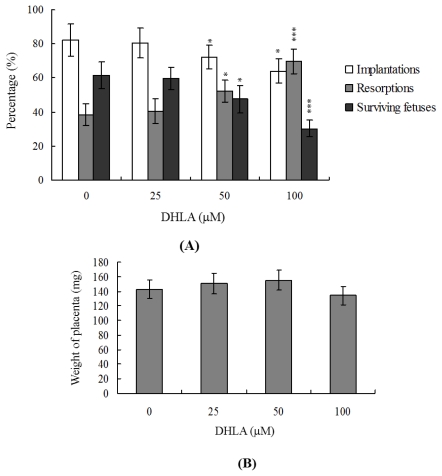

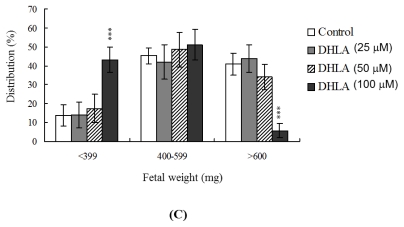
Effects of DHLA on *in vivo* implantation, resorption, fetal survival and fetal weights of mouse blastocysts. (**A**) Mouse blastocysts were treated with DHLA (25, 50 or 100 μM) for 24 h or left untreated. Implantations, resorptions and surviving fetuses were analyzed, as described in Materials and Methods. The percentage of implantations represents the number of implantations per number of transferred embryos × 100. The percentage of resorptions or surviving fetuses signifies the number of resorptions or surviving fetuses per number of implantations × 100; (**B**) Placental weights of 40 recipient mice were measured; (**C**) Weight distribution of surviving fetuses on day 18 post-coitus. Surviving fetuses were obtained by embryo transfer of control and DHLA-pretreated blastocysts, as described in Materials and Methods (320 total blastocysts across 40 recipients). * *P* < 0.05 and *** *P* < 0.001 *versus* the DHLA-free group.

To determine the effects of DHLA on blastocyst development *in vivo*, we transferred control and DHLA-pretreated mouse blastocysts, and examined the uterine content at 13 days post-transfer (day 18 post-coitus). The implantation ratios in the 50–100 μM DHLA-pretreated groups were significantly lower than that of the untreated control group (Figure 4A). Embryos that implanted but failed to develop were subsequently resorbed. However, the proportion of implanted embryos that failed to develop normally was markedly higher in the group treated with 50–100 μM DHLA (Figure 4A). Interestingly, no significant differences in placental weight were observed between the DHLA-treated and untreated groups (Figure 4B). However, fetal weight was lower in the 100 μM DHLA-treated group compared to the control group (479 ± 61 mg *versus* 611 ± 68 mg, respectively). Previous studies, including a recent report by our group, showed that 35–40% of fetuses weigh more than 600 mg, and the average weight of total surviving fetuses is about 600 ± 12 mg in the untreated control group at day 18 of pregnancy in a mouse embryo transfer assay [23,27–30]. Fetal weight is an important indicator of developmental status, and the average fetal weight of the untreated control group is used as a key marker of development of blastocysts treated with 100 μM DHLA. Interestingly, only 5.8% of the fetuses in the 100 μM DHLA-pretreated group weighed more than 600 mg (indicative of successful embryonic and fetal development), whereas 43% of control fetuses exceeded this threshold (Figure 4C). These observations collectively indicate that exposure to high concentrations of DHLA (such as 100 μM) at the blastocyst stage reduces embryo implantation and the potential for post-implantation development.

### 2.5. Disruption of Blastocyst Development by DHLA *in Vivo*

**Figure 5:**
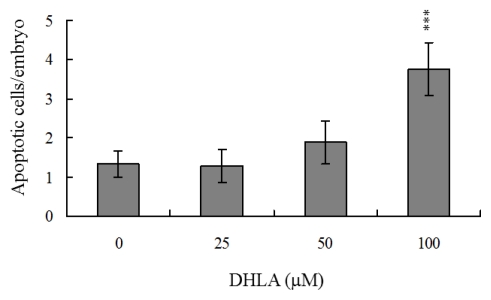

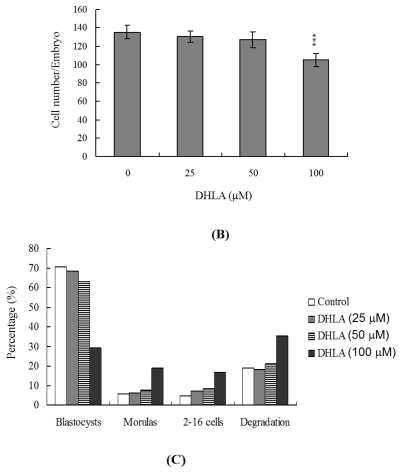
Effects of dietary DHLA on apoptosis and blastocyst development in an animal model. For the duration of the experiment, randomly selected female mice were fed a standard diet and drinking water supplemented with or without DHLA (25, 50 or 100 μM). After 24 h, female mice were mated overnight with a single fertile male of the same strain. Blastocysts were obtained by flushing the uterine horn on day 4 after mating. (**A**) Apoptosis of mouse blastocysts was examined by TUNEL staining followed by light microscopy, and the mean number of apoptotic (TUNEL-positive) cells per blastocyst calculated; (**B**) The total numbers of cells per blastocyst were counted; (**C**) Embryos obtained from mouse uterine horns on day 4 were examined for comparison of the developmental stages. Data are presented as a percentage of total embryos. Values are presented as means ± SEM (*n* = 6). *** *P* < 0.001 *versus* untreated control group.

Next, we examined the effects of DHLA on blastocyst development in an animal model. Female mice were fed a standard diet and drinking water supplemented with or without DHLA (25, 50 or 100 μM). DHLA consumption led to significant apoptosis and decreased cell proliferation in mouse blastocysts (Figure 5A,B). In addition, DHLA inhibited embryonic development to the blastocyst stage, causing frequent termination at the 2–16 cell or morula stage or degradation (Figure 5C). These results further validate the theory that exposure to high concentrations of DHLA (50–100 μM) through intake is potentially hazardous for mouse embryonic development.

## 3. Discussion

During the complex and precisely orchestrated embryonic development process, chemical or physical injury can affect normal development and lead to malformation or miscarriage of the embryo. Thus, it is important to establish the possible teratogenic effects of various agents, including natural chemical compounds or capping ligands for the preparation of fluorescence nanoparticles, which could be developed as embryonic development tracers.

During normal embryogenesis, apoptosis (a unique morphological pattern of cell death) functions to clear abnormal or redundant cells in preimplantation embryos [31,32]. Apoptotic processes do not occur prior to the blastocyst stage during normal mouse embryonic development [33], and induction of apoptosis during the early stages of embryogenesis (*i.e.*, following exposure to a teratogen) causes embryonic developmental injury [22,23,28,34,35]. In the present study, we investigated whether DHLA adversely affects the blastocyst stage of mouse embryos and subsequent early pre- and post-implantation embryonic development. Preliminary data showed that DHLA treatment for 24 h induces apoptosis in mouse blastocysts (Figure 1). Based on this finding, we further analyzed the effects of DHLA on embryonic development by incubating blastocysts in medium containing 25, 50 or 100 μM DHLA for 24 h. DHLA treatment decreased cell viability in mouse blastocysts via apoptosis (Figures 1 and 2). Treatment of mouse blastocysts with 50 μM DHLA induced apoptosis, as evident from TUNEL staining data (Figure 1). Dual differential staining results further disclosed that DHLA-induced cell loss and apoptosis occurs primarily in the ICM (Figure 2).

The TE arises from the trophoblast at the blastocyst stage and develops into a sphere of epithelial cells surrounding the ICM and blastocoel. These cells contribute to the placenta, and are required for development of the mammalian conceptus [36]. Thus, reduction in the TE cell lineage may suppress implantation and embryonic viability [37,38]. However, in our experiments, DHLA induced cell apoptosis specifically in the ICM and not TE, indicative of deleterious effects on embryonic development *in vitro*, implantation and post-implantation development *in vivo* or disruption of blastocyst development in an animal model (Figures 2–5). Previous studies have reported a reduction of at least ~30% in the number of cells in the ICM, associated with high risk of fetal loss or developmental injury, even in cases where the implantation rate and TE cell numbers are normal [39]. In addition, the ICM cell number is essential for proper implantation, and reduction in the cell lineage may decrease embryonic viability [37,38]. Our observation that DHLA treatment reduced the cell number and promoted apoptosis in the ICM of mouse blastocysts, but had no effect on the TE (Figure 2), led us to investigate the possibility that DHLA induces mortality and/or developmental delay in post-implantation mouse embryos *in vitro* and *in vivo*. DHLA-treated blastocysts displayed decreased embryonic development and increased embryonic death *in vitro* and reduced implantation *in vivo* (Figures 3 and 4). Our study results obtained using an animal assay model signify that DHLA exposure through dietary intake has the potential to cause hazardous effects on mouse embryonic development (Figure 5). These study results imply that development and preparation of fluorescence nanoparticles using DHLA as a capping ligand has a latent ability to cause cytotoxicity and injury in embryonic development.

## 4. Experimental Section

### 4.1. Materials

Pregnant mare’s serum gonadotropin (PMSG), Bovine serum albumin (BSA), sodium pyruvate and dihydrolipoic acid were purchased from Sigma (St. Louis, MO, USA). Human chorionic gonadotropin (hCG) was obtained from Serono (NV Organon Oss, the Netherlands). The TUNEL *in situ* cell death detection kit was obtained from Roche (Mannheim, Germany) and CMRL-1066 medium was from Gibco Life Technologies (Grand Island, NY, USA).

### 4.2. Collection of Mouse Morulas and Blastocysts

ICR mice were from National Laboratory Animal Center (Taiwan). This research was also approved by the Animal Research Ethics Board of Chung Yuan Christian University (Taiwan). All animals received humane care, as outlined in the Guidelines for Care and Use of Experimental Animals (Canadian Council on Animal Care, Ottawa, 1984). All mice were maintained on breeder chow (Harlan Teklad chow) with food and water available *ad libitum*. Housing was in standard 28 cm × 16 cm × 11 cm (height) polypropylene cages with wire-grid tops and kept under a 12 h·day/12 h night regimen. Nulliparous females (6–8 weeks old) were superovulated by injection of 5 IU PMSG followed 48 h later by injection of 5 IU hCG, and then mated overnight with a single fertile male of the same strain. The day a vaginal plug was found was defined as day 0 of gestation. Plug-positive females were separated for experimentation. Morulas were obtained by flushing the uterine tubes on the afternoon of gestation day 3, and blastocysts were obtained by flushing the uterine horn on day 4; in both cases the flushing solution consisted of CMRL-1066 culture medium containing 1 mM glutamine and 1 mM sodium pyruvate. The concentration of glucose in this medium was 5 mM. Expanded blastocysts from different females were pooled and randomly selected for experiments.

### 4.3. DHLA Treatment and TUNEL Assay

Blastocysts were incubated in medium containing the indicated concentrations of DHLA for 24 h. For apoptosis detection, embryos were washed in DHLA-free medium, fixed, permeabilized and subjected to TUNEL labeling using an *in situ* cell death detection kit (Roche Molecular Biochemicals, Mannheim, Germany) according to the manufacturer’s protocol. Photographic images were taken under bright field illumination using a fluorescence microscope.

### 4.4. DHLA Treatment and Cell Proliferation

Blastocysts were incubated with or without culture medium containing 0, 25, 50 or 100 μM DHLA. After 24 h they were washed with DHLA-free medium and dual differential staining was used to facilitate counting of cell numbers in the inner cell mass (ICM) and trophectoderm (TE) [37]. Blastocysts were incubated in 0.4% pronase in M_2_-BSA medium (M_2_ medium containing 0.1% bovine serum albumin) for removal of the zona pellucida. The denuded blastocysts were exposed to 1 mM trinitrobenzenesulphonic acid (TNBS) in BSA-free M_2_ medium containing 0.1% polyvinylpyrrolidone (PVP) at 4 °C for 30 min, and then washed with M_2_ medium [40]. The blastocysts were further treated with 30 μg/mL anti-dinitrophenol-BSA complex antibody in M_2_-BSA at 37 °C for 30 min, and then with M_2_ medium supplemented with 10% whole guinea-pig serum as a source of complement, along with 20 μg/mL bisbenzimide and 10 μg/mL propidium iodide (PI), at 37 °C for 30 min. The immunolysed blastocysts were gently transferred to slides and protected from light before observation. Under UV light excitation, the ICM cells (which take up bisbenzimidine but exclude PI) appeared blue, whereas the TE cells (which take up both fluorochromes) appeared orange-red. Since multinucleated cells are not common in preimplantation embryos [41], the number of nuclei was considered to represent an accurate measure of the cell number.

### 4.5. Annexin V Staining

Blastocysts were incubated in 25, 50 or 100 μM DHLA for 24 h, washed with DHLA-free culture medium, and then stained using an Annexin V-FLUOS staining kit (Roche), according to the manufacturer's instructions. Briefly, the blastocysts were incubated in M_2_-BSA for removal of the zona pellucida, washed with PBS plus 0.3% BSA, and then incubated for 60 min with a mixture of 100 μL binding buffer, 1 μL fluorescein isothiocyanate (FITC)-conjugated Annexin V and 1 μL PI. After incubation, the embryos were washed and photographed using a fluorescence microscope under fluorescent illumination. Cells staining Annexin V+/PI− were considered apoptotic, while those staining Annexin V+/PI+ were considered necrotic.

### 4.6. Morphological Analysis of Embryonic Development

Blastocysts were cultured according to a modification of the previously reported method [42]. Briefly, embryos were cultured in 4-well multidishes at 37 °C. For group culture, four embryos were cultured per well. The basic medium consisted of CMRL-1066 supplemented with 1 mM glutamine and 1 mM sodium pyruvate plus 50 IU/mL penicillin and 50 mg/mL streptomycin (hereafter called culture medium). For treatments, the embryos were cultured with the indicated concentrations of DHLA for 24 h in serum-free medium. Thereafter, the embryos were cultured for 3 days in culture medium supplemented with 20% fetal calf serum, and for 4 days in culture medium supplemented with 20% heated-inactivated human placental cord serum, for a total culture time of 8 days from the onset of treatment. Embryos were inspected daily under a phase-contrast dissecting microscope, and developmental stages were classified according to established methods [43,44]. Under these culture conditions, each hatched blastocyst attached to the fibronectin and grew to form a cluster of ICM cells over the trophoblastic layer via a process called TE outgrowth. After a total incubation period of 96 h, morphological scores for outgrowth were estimated. Growing embryos were classified as either “attached” or “outgrowth”, with the latter defined by the presence of a cluster of ICM cells over the trophoblastic layer. As described previously [45], ICM clusters were scored according to shape, ranging from compact and rounded ICM (+++) to a few scattered cells (+) over the trophoblastic layer.

### 4.7. Blastocyst Development Following Embryo Transfer

To examine the ability of expanded blastocysts to implant and develop *in vivo*, the generated embryos were transferred to recipient mice. ICR females (white skin color) were mated with vasectomized males (C57BL/6J; black skin color; from National Laboratory Animal Center, Taiwan, ROC) to produce pseudopregnant dams as recipients for embryo transfer. To ensure that all fetuses in the pseudopregnant mice came from embryo transfer (white color) and not from fertilization by C57BL/6J (black color), we examined the skin color of the fetuses at day 18 post-coitus. To assess the impact of DHLA on postimplantation growth *in vivo*, blastocysts were exposed to 0, 25, 50 and 100 μM DHLA for 24 h, and then 8 embryos were transferred in parallel to the paired uterine horns of day 4 pseudopregnant mice. The surrogate mice were killed on day 18 post-coitus, and the frequency of implantation was calculated as the number of implantation sites per number of embryos transferred. The incidence rates of resorbed and surviving fetuses were calculated as the number of resorptions or surviving fetuses, respectively, per number of implantations. The weights of the surviving fetuses and placentae were measured immediately after dissection.

### 4.8. Statistics

The data were analyzed using one-way ANOVA and *t*-tests and are presented as the mean ± SEM, with significance at *P* < 0.05.

## 5. Conclusions

Based on these results, we propose that development and preparation of fluorescence nanoparticles using DHLA as a capping ligand has a latent ability to cause cytotoxicity and injury in embryonic development. However, it appears that concentrations of DHLA lower than 50 μM do not exert a hazardous effect on embryonic development. Moreover, our previous study demonstrated that at doses of less than 50 μM (0–25 μM), DHLA does not exert hazardous effects on ESC-B5 cell properties, including viability, development and differentiation. These findings imply that the DHLA content is critical to determine whether or not a particular nanoparticle type is suitable as an *in vitro* or *in vivo* embryonic development tracer.
